# Collectanea Medica, Consisting of Anecdotes, Facts, Extracts, Illustrations, Queries, Suggestions, &c.

**Published:** 1816-06

**Authors:** 


### COLLECTANEA MEDICA,

CONSISTING OF

### ANECDOTES, FACTS, EXTRACTS, ILLUSTRATIONS, QUERIES, SUGGESTIONS, &c.

RELATING TO THE

*History or the Art of Medicine, and the Auxiliary Sciences.*

Quicquid agunt medici,

Nostri farrago libelli.

THE attention of naturalists, and more particularly those connected with medicine, has been lately much attracted to parasite animalcules, especially such as live in the human body. A case lately occurred, at one of the City Prisons, of a man so infested with lice all over his body, that he was suspected to be afflicted with the morbus pedicularis. Dr. Adams procured some of the lice, which Sir Joseph Banks requested Dr. Leech, of the British Museum, to examine with peculiar diligence, in order to ascertain whether they differed at all from the common body-louse. After a most minute comparison, Dr. L. ascertained, beyond all question, that they are the same. At present, therefore, no other pediculi are known whose *habitat* is on the human body, excepting the common or head louse, the crab-louse or *pediculus inguinalis*, and the body-louse. We have, in a few late Numbers of our Journal, entered a little into the controversy concerning the *Acarus Syro*, or *A. Scabiei*, as it is sometimes called, and given Dr. Adams's reasons for asserting that such an insect has never been found in the true itch; but that it excites a disease peculiar to itself, and which may be cured by a diligent attention to the extraction of the acarus. We now offer the following passage, extracted from a popular work, which we rather prefer, as few of the faculty have opportunities of examining the question in all its scientific array, or even in a practical form.

### *Extract from Messrs. Kirby and Spence's Introduction to Entomology.*

Insects, as to their *direct* attacks upon us, may be arranged in three principal classes. Those, namely, which seek to make us their food; those whose object is to prevent or revenge an injury which they either fear, or have received from us; and those which indeed offer us no violence, but yet incommode us extremely in other ways.

I hope I shall not too much offend your delicacy if I begin the first class of our insect assailants with a very disgusting genus, which Providence seems to have created to punish inattention to personal cleanliness. But, though this pest of man must not be wholly wholly passed over, yet, since it is unfortunately too well known, it will not be at all necessary for me to enlarge upon its history. I shall only mention one fact, which shows the astonishingly rapid increase of these animals, where they have once gotten possession. It is a vulgar notion, that a louse in twenty-four hours may see two generations; but this is rather overshooting the mark. Leeuwenhoek, whose love for science overcame the nausea that such creatures are apt to excite, proves that their nits or eggs are not hatched till the eighth day after they are laid, and that they do not themselves commence laying before they are a month old. He ascertained, however, that a single female louse may, in eight weeks, witness the birth of five thousand descendants.* You remember how wolves were extirpated from this country, but, perhaps, never suspected any monarch of imposing a tribute of *lice* upon his subjects. Yet we are gravely told that in Mexico and Peru such a *poll*-tax was exacted, and that bags full of these treasures were found in the palace of Montezuma!!!† Were our own taxes paid in such coin, what little grumbling would there be!

Two other species of this genus, besides the common louse, are, in this country, parasites upon the human body——But already I seem to hear you exclaim, "Why dwell so long on creatures so odious and nauseating, whose injuries are confined to the *profanum vulgus?* Leave them, therefore, to the canaille—they are nothing to us." Not so fast, my friend—recollect what historians and other writers have recorded concerning the *Phthiriasis* or pedicular disease, and you must own that, for the quelling of human pride, and to pull down the high conceits of mortal man, this most loathsome of all maladies, or one equally disgusting, has been the inheritance of the rich, the wise, the noble, and the mighty; and, in the list of those that have fallen victims to it, you will find poets, philosophers, prelates, princes, kings, and emperors. It seems more particularly to have been a judgment of God upon oppression or tyranny, whether civil or religious. Thus the inhuman Pheretima mentioned by Herodotus, Antiochus Epiphanes, the Dictator Sylla, the two Herods, the Emperor Maximian, and, not to mention more, the great persecutor of the Protestants, Philip the Second, were carried off by it.

I say by this malady, *or one equally disgusting*, because it is not by any means certain, though some learned men have so supposed, that all these instances, and others of a similar nature, standing also upon record, are to be referred to the same specific cause; since there is very sufficient reason for thinking that at least *three* different descriptions of insects are concerned in the various cases that have been handed down to us under the common name

* Leeuw. Epist. 98. 1696.

† Bingley, Anim. Biogr. first edition, iii. 437. St. Pierre's Studies, &c. i. 312.

of *Phthiriasis.* As the subject of maladies connected with insects, or produced by them, is both curious and interesting, although no writer, that I am aware of, has given it full consideration, and at the same time falls in with my general design, I hope you will not regard me as guilty of presumption, and of intruding into the province of medical men, if I enter rather largely into it, and state to you the reasons that have induced me to embrace the above hypothesis, leaving you at full liberty to reject it if you do not find it consonant to reason and fact. The three kinds of insects to which I allude, as concerned in cases that have been deemed Phthiriasis, are *Pediculi*, *Acari*, and *Larvæ* in general.

As far as the habits of the genus *Pediculus*, whether inhabiting man or the inferior animals, are at present known, it does not appear, from any well ascertained fact, that the species belonging to it are ever *subcutaneous.* For this observation, as far as it relates to man, I can produce the highest medical authority. "The louse feeds on the surface of the skin," says the learned Dr. Mead, in his Medica Sacra; and Dr. Willan, in his palmary work on Cutaneous Diseases, remarks, with respect to the body-louse, "that the nits, or eggs, are deposited on the small hairs of the skin," and that "the animals are found on the skin, or on the linen, and not under the cuticle, as some authors have represented." And he further observes, that "many marvellous stories are related by Forestus, Schenkius, and others, respecting lice bred under the skin, and discharged in swarms from abscesses, strumous ulcers, and vesications. The mode in which Pediculi are generated being now so well ascertained, no credit can be given to these accounts." Thus far this great man, who, however, supposes (in which opinion Dr. Bateman concurs with him) that the authors to whom he alludes had mistaken for lice some other species of insects, which are not unfrequently found in putrefactive sores.

If these observations be allowed their due weight, it will follow, that a disease produced by animals residing under the cuticle cannot be a true Phthiriasis, and therefore the death of the poet Alcman, and of Pherecydes Syrius the philosopher, mentioned by Aristotle, must have been occasioned by some other kind of insect. For, speaking of the lice to which he attributes these catastrophes, he says that "they are produced in the flesh in small pustule-like tumours, which have no pus, and from which, when punctured, they issue."* For the same reason, the disorder which Dr. Heberden has described in his Commentaries, from the communications of Sir E. Wilmot, under the name of *Morbus Pedicularis*, must also be a different disease, since, with Aristotle, he likewise represents the insects as inhabiting tumours, from which they may be extracted when opened by a needle. He says, indeed, that in every respect they resemble the common lice, except in being

* Hist. Animal. l. 5. c. 31.

whiter; whiter; but medical men, who were not at the same time entomologists, might easily mistake an Acarus for a Pediculus.*

Dr. Willan, in one case of *Prurigo senilis,* observed a number of small insects on the patient's skin and linen. They were quick in their motion, and so minute that it required some attention to discover them. He took them at first for small Pediculi; but under a lens they appeared to him rather to be a nondescript species of Pulex;† yet the figure he gives has not the slightest likeness to the latter genus, while it bears a striking resemblance to the former. It is not clear whether his draughtsman meant to represent the insect with six or with eight legs: if it had only six, it was probably a Pediculus; but if it had eight, it would form a new genus between the *Acaridæ* and the hexapod *Aptera.* Dr. Bateman, in reply to some queries put to him, at my request, by our common and lamented friend Dr. Reeve, relates that he understood from Dr. Willan, in conversation, that the insect in question jumped in its motion. This circumstance he regards as conclusive against its being a Pediculus; but such a consequence does not necessarily follow, since it not seldom happens that insects of the same genus either have or have not this faculty; for instance, *Cyphon hemisphœricus Acarus scabiei*, &c.

Dr. Willan has quoted with approbation two cases from Amatus Lusitanus, which he seems to think correctly described as Phthiriasis. In one of them, however, which terminated fatally, the circumstances seem rather hyperbolically stated—I mean, where it is said that two black servants had no other employment than carrying baskets full of these insects to the sea!! Perhaps you will think I draw largely upon your credulity, if I call upon you to believe this; I shall therefore leave you to act as you please. Thus much for pure Phthiriasis, which term ought to be confined to maladies produced by *lice.*—I shall only further observe, that, as many species as exist of these, which are the causes of disease, so many kinds of Phthiriasis will there be.

*Acari,* or mites, are the next insect sources of disease in the human species, and that not of one, but probably of many kinds, both local and general. They are distinguished from Pediculi not only by their form, but also often by their situation, since they frequently establish themselves under the cuticle. With respect to local disorders, Dr. Adams conjectures that Acari may be the cause of certain cases of ophthalmia. Sir J. Banks, in a letter to that gentleman, relates that some seamen belonging to the Endeavour brig, being tormented with a severe itching round the extremities of the eye-lids, one of them was cured by an Otaheitan

* From the terms employed by Aristotle and Dr. Mead in their Account of these cases, it does not appear that the animal they meant could be a maggot, but something bearing a more general resemblance to lice.

† On Cutaneous Diseases, 87, 88; and t. 7. f. 4.

woman, woman, who, with two small splinters of bamboo, extracted from between the *ciliæ* abundance of very minute lice, which were scarcely visible without a lens, though their motion, when laid on the thumb, was distinctly perceived. These insects were probably synonymous with the *Ciron des paupières* of Sauvages.*—Le Jeune, a French physician quoted in Mouffet, describes a case, in which what seems a different species, since he calls them rather large, infested the white of the eye, exciting an intolerable itching.† Dr. Mead, from the German Ephemerides, gives an account of a woman suckling her child, from whose breast proceeded very minute vermicles.‡ These were probably Acari, and perhaps that species which, from its feeding upon milk, Linné denominates *A. Lactis.* The great author last-mentioned describes an insect, a native of America, under the name of *Pediculus ricinoides*, which, upon the authority of Rolander, he informs us gets into the feet of people as they walk, sucks their blood, oviposits§ in them, and so occasions very dangerous ulcers. It would be an Acarus, he observes, but it has only six legs. Now Herman affirms, that some species of *Trombidium* (a genus separated by Fabricius from *Acarus)* have in no state more than six legs.‖ Others of the tribe of *Acaridæ*, and the insect in question amongst the rest, may be similarly circumstanced; or those that Rolander examined might have been larvæ, which in this tribe are usually hexapods.

Linné appears to have been of opinion that many contagious diseases are caused by Acari.¶ How far he was justified in this opinion I shall not here inquire; facts alone can decide the question, and observations made by men acquainted with entomology as well as the science of diseases. Considerable deference and attention, however, are certainly due to the sentiments of so great a naturalist, in whom these necessary qualifications were united in no common degree. With respect to the dysentery and the itch, he affirms that this had been manifested to his eyes. You will wish probably to know the arguments that may be adduced in confirmation of this opinion; I will therefore endeavour to satisfy you as well as I am able. The following history, given by Linné, seems to prove the dysentery connected with Acari.

Rolander, a student in entomology, while he resided in the house

---

* On Morbid Poisons, 306, 307.

† Mouffet, 267.

‡ Medica Sacra, 104, 105.

§ It is to be hoped this new word may be admitted, as the *laying of eggs* cannot otherwise be expressed without a periphrasis. For the same reason its substantive *oviposition* will be employed.

‖ Mem. apterologique, 19.

¶ Insecta ejusmodi minutissima, forte *acaros* diversæ speciei causas esse diversorum morborum contagiosorum, ab analogia et experientia hactenus acquisita, facili credimus negotio. *Amœn. Ac.* v. 94.

of of the illustrious Swede, was attacked by the disease in question, which quickly gave way to the usual remedies. Eight days after it returned again, and was, as before, soon removed. A third time, at the end of the same period, he was seized with it. All the while he had been living like the rest of the family, who had nevertheless escaped. This, of course, occasioned no little inquiry into the cause of what had happened. Linné, aware that Bartholinus had attributed the dysentery to *insects*, which he professed to have seen, recommended it to his pupil to examine his fæces. Rolander, following this advice, discovered in them innumerable animalcules, which, upon a close examination, proved to be Acari. It was next a question how he alone came to be singled out by them; and thus he accounts for it. It was his habit not to drink at his meals; but, in the night, growing thirsty, he often sipped some liquid out of a vessel made of juniper wood. Inspecting this very narrowly, he observed, in the chinks between the ribs, a white line, which, when viewed under a lens, he found to consist of innumerable Acari, precisely the same with those that he had voided. Various experiments were tried with them, and a preparation of rhubarb was found to destroy them most effectually. He afterwards discovered then in vessels containing acids, and often under the bung of casks.* In the instance here recorded, the dysentery, or diarrhœa, was evidently produced by these Acari; but it would be going too far, I apprehend, to assert that they are invariably the cause of that disease.

That *Scabies*, or the itch, is occasioned by an Acarus, is not a doctrine peculiar to the moderns. Mouffet mentions Abinzoar, called also Avenzoar, a celebrated Hispano-Arabian physician of Seville, who flourished in the twelfth century, as the most ancient author that notices it. He calls these Acari little lice that creep under the skin of the hands, legs, and feet, exciting pustules full of fluid.† Joubert, quoted by the same author, describes them under the name of *Sirones*, or mites, as always being concealed beneath the epidermis, under which they creep like moles, gnawing it, and causing a most troublesome itching. It appears that Mouffet, or whoever was the author of that part of the *Theatrum Insectorum*, was himself also well acquainted with these animals, since he remarks that their habitation is not in the pustule but near it. A remark afterwards confirmed by Linné,‡ and more recently by Dr. Adams.§ In common with the former of these authors, Mouffet further notices the effect of warmth upon them in exciting

---

* Amœn. Ac. v. 94–98.

† Mouffet, 266.

‡ Acarus sub ipsa pustula minimè quærendus est, sed longius recessit, sequendo rugam cuticulæ observatur. *Amœn. Ac.* v. 95. not. **.

§ Observations, &c. 296.

4 motion.

motion.* Our intelligent countryman also observes that they cannot be Pediculi, since they live under the cuticle, which lice never do.† In the epistle dedicatory, the editor speaks also of these Acari as living in burrows which they have excavated in the skin near a lake of water; from which if they be extracted with a needle, and put upon the nail, they show, in the sun, their red head and the feet with which they walk.‡ And, to close my *veteran* authorities, Junius thus explains the word Acarus, as I find him quoted in Gouldman's useful Dictionary, "A small worm, which eats under the skin, and makes burrows in itching hands."§

In more modern times, microscopical figures have been added to descriptions of the insect. Bonomo first furnished this valuable species of elucidation. His figures, however, which are copied by Baker in his work on the microscope, are far from accurate.‖ Those of De Geer and Dr. Adams are much more satisfactory, and mutually confirm each other.¶ From them it is evident that the same insect inhabits the scabies of Sweden and Madeira. Dr. Bateman, in the letter before alluded to, informs his correspondent that he had seen that from Madeira, and gives it as his opinion that there cannot be a doubt of the existence of an *Acarus Scabiei;* an opinion which he repeats in his late work on Cutaneous Diseases. From all this we may regard the point as so far settled, that such an animal exists at least as an occasional concomitant of scabies.

This fact being ascertained, a more complex inquiry remains, which branches out into two distinct questions. Is scabies always produced by these insects? or, if this be not the case, Is the *animate* scabies a distinct disease from the *inanimate?*

It is very remarkable that Linné, a physician as well as a naturalist, and De Geer, one of the most accurate observers that ever existed, should both assign the insect in question as the undoubted cause of the *common* scabies of their country; the one applying to

---

* Extractus aeu et super ungue positus, movet se si solis etiam calore adjuvetur. *ubi supr.* Ungui impositus vix movetur: si vero oris calido halitu affletur, agilis in ungue cursitat. *Fn. Suec.* 1975.

† Neque Syrones isti sunt de pediculorum genere, ut Joannes Langius ex Aristotele videtur asserere: nam illi extra cutem vivunt, hi vero non. *ubi supr.*

‡ Imo ipsi *acari* præ exiguitate indivisibiles, ex cuniculis prope aquæ lacum quos foderunt in cute, acu extracti et ungue impositi, caput rubrum, et pedes quibus gradiuntur ad solem produnt. p. vi.

§ Teredo sive exiguus vermiculus, qui subter cutim erodit agitque cuniculos in pruriginosis manibus. Gouldman tells us these *acari* were also called *hand-worms.* Another English name is given in Mouffet, viz. *wheale-worms.*

‖ Osservazioni intorno à pellicelli del corpo umano fatte dal Dottor Gio Cossimo Bonomo, &c. f. 1–3. Baker on Microsc. i. t. 13. f. 2.

¶ De Geer, vii. t. 5. f. 12–14.

the disease he was speaking of the epithet of *communissima*, and observing the fact to be notorious, *(cuique liquet,)* and the other designating it by its well-known French name "*La Gale.*"* And is it not equally remarkable that such men as John Hunter, Dr. Heberden, Dr. Bateman, Dr. Adams, and Mr. Baker, should never, in this country, have been able to meet with it? Did it indeed exist in our common scabies, it seems impossible that it could have escaped the observation of the two last of these gentlemen; Dr. Adams being so well qualified to detect it from his observations in Madeira, and Mr. Baker from his expertness in microscopical researches. Dr. Bateman, in the letter above quoted, says, "I have hunted it with a good magnifier, in many cases of itch, both in and near the pustules, and in the red streaks or furrows, but always without success." In his work on Cutaneous Diseases, he tells us, however, that he has seen it, in one instance, when it had been taken from the diseased surface by another practitioner. And, though Dr. Willan, in his book, speaks of the Acarus as the concomitant of this disease, yet his learned friend just mentioned observes, that he admitted that the insect was not to be found in ordinary cases, and indeed never seemed to have made up his mind upon the subject. When I was at Norwich last year (1812) Dr. Reeve very kindly accompanied me to the House of Industry there, to examine a patient whose body was very full of the pustules of this disorder; but, though we used a good magnifier, we could discover nothing like an insect. I must observe, however, that our examination was made in December, in severe weather, when the cold might, perhaps, render the animal torpid, and less easy to be discovered.

From the above facts, it seems fair to infer that this animal is not invariably the cause of scabies, but that there are cases with which it has no connection. Now, from this inference, would not another also follow, that the disease produced by the insect is specifically distinct from that in which it cannot be found? Sauvages and Dr. Adams are both of this opinion,† the former assigning to it the trivial name of *vermicularis;* and the latter proving, by very satisfactory arguments, that it is different from the other. If they were both *animate* diseases, but derived from two distinct species of animals, (for it seems not impossible that even our common itch may be caused by an Acarus more minute than the other, and so more difficult to find,) they would properly be considered as distinct species; much more, therefore, if one be *animate* and the other *inanimate*. Nay, this, I should think, would lead to a doubt

* I am informed by my learned friend Alexander Mac Leay, Esq. Secretary to the Linnæan Society, that, in the north of Scotland, the insect of the itch is well known, and easily discovered and extracted.

† This opinion Dr. Bateman thinks probably the true one. Cutan. Dis. 197.

whether even their *genus* were the same. I shall dismiss this part of my subject with the mention of a discovery of Dr. Adams, which seems to have escaped both Linné and De Geer—that the *Acarus Scabiei* is endowed with the faculty of leaping; (in this respect resembling the insect found by Willan in *Prurigo senilis* mentioned above,) for which purpose its four posterior thighs are incrassated.*

But, besides these *Acarine* diseases, there seems to be one (unless with Linné we regard the plague as of this class†) more fearful and fatal than them all. You will, perhaps, conjecture I am speaking of that described by Aristotle and Sir E. Wilmot as the Phthiriasis, and your conjecture will be right. But some think, and those men of merited celebrity, that *Acari* have nothing to do in these and similar cases, for that *maggots* were the parasites mistaken for lice. This, from the passage above quoted, appears to have been Dr. Willan's opinion ; it was also Professor Murray's, to which, in the letter so often referred to, Dr. Bateman subscribes, adding, as a reason for excluding Acari from being concerned, that "they are too minute, and never have been seen in such numbers as to be mistaken for lice." But both Acari and Pediculi vary in size, some of the former being larger than some of the latter. And allowing them to be ever so minute, yet when they issue in swarms, as mites from a cheese, they would be very visible, were it only from their motion. Besides, as they are furnished with legs, their motions resemble those of lice infinitely more than do the contortions of maggots. So that an Acarus would be deemed a louse much sooner by an unentomological observer than would a maggot. Whether Acari have ever been seen in such numbers as to be mistaken for lice, is the point in question; and therefore, by itself, cannot be admitted for a valid argument. Though *Acarus Scabiei* does not appear to swarm in ordinary cases, yet this is certainly no reason why other species may not do so. Where it has once made a settlement, how incredibly, and in how short a space of time, does the *Siro* or cheese-mite multiply! *Acarus destructor*, and many other species, are equally rapid in their increase.

I shall now produce two instances where Acari were evidently concerned. Dr. Mead, from the *German Ephemerides*, relates the miserable case of a French nobleman, from whose eyes, nostrils, mouth, and urinary passage, animalcules, of a red colour, and excessively minute, broke forth day and night, attended by the most horrible and excruciating pains, and at length occasioning his death. The account further says, that they were produced from his corrupted blood. This was probably a fancy originating in their red colour; but the whole history, whether we consider the

* Probably this Acarus, in the modern system, would form a distinct genus. Latreille places it in his *Sarcoptes* with the *Ac. passerinus*, L. Latr. *Gen.* i. 152. 2.

† *Amœn. Ac.* ubi. supr. 101.

size size and colour of the animals, or the places from which they issue, is inapplicable to *larvæ* or maggots, and agrees very well with *Acari*, some of which, particularly *A. autumnalis*, are of a bright red colour. The other case, and a very similar one, is that recorded by Mouffet of Lady Penruddock; concerning whom he expressly tells us, that Acari swarmed in every part of her body—her head, eyes, nose, lips, gums, the soles of her feet, &c., tormenting her day and night, till, in spite of every remedy, all the flesh of her body being consumed, she was at length relieved by death from this terrible state of suffering. Mouffet attributes her disease to the *Acarus Scabiei;* but, from the symptoms and fatal result, it seems to have been a different and much more terrific animal. He supposes, in this instance, the insect to have been generated by drinking goat's milk too copiously. This, if correct, would lead to a conjecture that it might have been the *A. lactis*, L.

---

The following passage, from the Abbé Raynal's History of the European Settlements in the East and West Indies, published at least forty years ago, will not prove uninteresting to our readers. Though it may not be correct in every point, it is sufficient to show that the lately revived custom of frequent bleeding in the early stage of yellow fever was the common practice till unhappily superseded by the doctrine, or rather the language, of typhus.

"The Americans seem to have been destined by nature to a greater share of happiness than the Europeans. In the islands are scarcely known such diseases as the gout, gravel, stone, apoplexies, pleurisies, complaints of the chest, and other disorders, which winter occasions: None of those scourges of the human race, which are so fatal in other countries, have ever made the least ravages there. If the air of the country can be withstood, and the middle age be attained to, this is sufficient to insure a long and happy course of life. There, old age is not tottering, languishing, and beset with those infirmities which affect it in our climate.

"In the Caribbees, however, new-born infants are attacked with a disease which seems peculiar to the torrid zone: it is called *tetanos*. If a child receives the impressions of the air or wind, if the room where it is just born is exposed to smoke, to too much heat or cold, the disorder shows itself immediately. It begins by seizing the jaw, which becomes rigid and fixed, so as not to be opened. This spasm soon communicates itself to the other parts of the body; and the child dies for want of being able to take nourishment. If it escapes this danger, which threatens the nine first days of its existence, it has nothing to fear. The indulgences which are allowed to children before they are weaned, which is at the end of twelve months, such as the use of coffee, chocolate, wine, but especially sugar and sweetmeats; these indulgencies, that are so pernicious to our children, are offered to those of

 America

America by nature, which accustoms them in early age to the productions of their climate.

The fair sex, naturally weak and delicate, has its infirmities as well as its charms. In the islands, they are subject to a weakness; an almost total decay of their strength; an unconquerable aversion for all kind of wholesome food, and an irregular craving after every thing that is prejudicial to their health. Salt or spiced food is what they only relish and desire. This disease is a true cachexy, which commonly degenerates into a dropsy. It is attributed to the diminution of the catamenia in those women who come from Europe, and to the weakness or total suppression of that periodical discharge in Creoles.

" The men, more robust, are liable to more violent complaints. In this vicinity of the equator, they are exposed to a hot and malignant fever, known under different names, and indicated by hæmorrhages. The blood, which is boiling under the fervent rays of the sun, is discharged from the nose, eyes, and other parts of the body. Nature, in temperate climates, does not move with such rapidity, but that, in the most acute disorders, there is time to observe and follow the course she takes. In the islands, her progress is so rapid, that, if we delay to attack the disorder as soon as it appears, its effects are fatal. Thus it is that the patient, in the space of twenty-four hours, must be bled fifteen or eighteen times, while, in the intervals, he has recourse to other remedies. No sooner is a person seized with sickness, but the physician, the lawyer, and the priest, are all called to his bed-side.

" Most of those who survive these violent shocks, being exhausted by the manner in which they have been treated, recover very slowly, and with difficulty. Several fall into an habitual weakness, occasioned by the debility of the whole machine, which the noxious air of the country, and the little nourishment their food supplies, are not able to restore. Hence obstructions, jaundice, and swelling of the spleen, are produced, which sometimes terminate in dropsies.

" Almost all the Europeans who land in America are exposed to this danger, and frequently the Creoles themselves, on their return from more temperate climates. But it never attacks women whose blood has the natural evacuations; and negroes, who, born under a hotter climate, are inured by nature, and prepared by a free perspiration, for all the ferments that the sun can produce.

" It is certainly owing to the sun, the heat of whose rays, being less oblique, and more constant than in our climates, occasions these violent fevers. Its heat must inevitably produce a thickening of the blood, through the excess of perspiration, a want of elasticity in the solids, a dilatation of the vessels by the impulse of the fluids, whether in proportion to the rarefaction of the air, or the less degree of compression which the surface of the bodies is exposed to in a rarefied atmosphere.

" Some of these inconveniencies might, perhaps, be prevented, by purging and bleeding on the passage, as we advance toward the torrid torrid zone, by repeating these precautions in the islands, and by the use of the cold bath.

"But, far from having recourse to these expedients, which reason indicates, the inhabitants fall into such excesses, as are most likely to hasten and increase the disorder. The strangers who arrive at the Caribbees, excited by the entertainments they are invited to, the pleasures they partake of, and the kind reception they meet with, give themselves up to an immoderate indulgence of all the pleasures which custom renders less prejudicial to those who are born under this climate. Feasting, dancing, gaming, late hours, wines, cordials, and frequently the chagrin of disappointment in their sanguine expectations, conspire to add to the ferment of an immoderate heat of the blood, which soon becomes inflamed.

"With such indulgence, it is scarce possible to resist the heats of this climate; and even the greatest precautions are not sufficient to secure persons from the attack of those dangerous fevers; seeing the most sober and moderate men, who are the most averse to every kind of excess, and the most careful in all their actions, are victims to the new air they breathe. In the present state of the colonies, of ten men that go into the islands, four English die, three French, three Dutch, three Danes, and one Spaniard.

"When it was observed how many men were lost in these regions, at the time they were first occupied, it was generally thought, that the states who had the ambition of settling there, would be depopulated in the end."

---

We extract the following article from the last volume of the Transactions of the London College of Physicians; and cannot help remarking, that, had a similar case occurred in vaccination under a less attentive practitioner, it might have passed for an unsuccessful instance of the prophylactic power of that operation.

*A Case of Natural Small-pox, occurring several Years after Inoculation with Variolous Matter; in consequence of the Progress of the inoculated Pustules being interrupted.* By Mr. Millington.

Mr. L———'s child, of Castle-street, Oxford Market, was inoculated by me with small-pox matter; and, at the same time, and with the same sort of matter, I inoculated one of his lodger's children. At the period of four days, the punctures, two in number, on one arm, appeared a little raised and inflamed, which insured that I should have the proper effect produced. On the ninth day after the insertion of the matter, I called on the child, and observed to the parents that the pustules on the arm had been interfered with, for they were burst: they were quite unacquainted with the cause, but supposed the child had scratched the arm in the night. I saw the child the following day, and inquired if she had been ill; they did not know that she had been so, but were doubtful. No eruption followed, and the child was not, as I informed the parents, secured from future infection from small-pox. But they were were of the opinion that the child had the disease satisfactorily, and would not consent to have it re-inoculated. The lodger's child, that was inoculated at the same time with the aforesaid child, did not appear to be affected by the matter till after the period of 16 or 18 days, about which time the incisions took on inflammation, and produced the symptomatic fever, as also a plentiful crop of eruptions, that continued about a week, and went off in the usual manner. Several years after this transaction, the father of the child that had been inoculated, and that had not had any eruption or fever, called on me one evening about dusk, and begged me to go with him to see his child, that was at that time extremely unwell, with fever and delirium. When I saw the child, I inquired if she had ever had the small-pox; he smiled, and said I had inoculated her some years before with the small-pox. I then recollected the circumstance, and gave it as my opinion, that the eruptions that were then coming out were small-pox. The next day it was put beyond all doubt; the child had a heavy crop of pustules, and suffered considerably during the disease, but recovered very well.

